# Supplementary material for: Deep-learning-based gene perturbation effect prediction does not yet outperform simple linear baselines
Source: Nat Methods. 2025 Aug 4;22(8):1657–61. doi: 10.1038/s41592-025-02772-6 (PMC12328236; doi:10.1038/s41592-025-02772-6)
Supplement: Supplementary file 1 — Reporting Summary [file 41592_2025_2772_MOESM1_ESM.pdf]

Corresponding author(s): *Double-anonymous peer review submissions: write DAPR and your manuscript number here instead of author names.*

Last updated by author(s): *YYYY-MM-DD*

Reporting Summary

Nature Portfolio wishes to improve the reproducibility of the work that we publish. This form provides structure for consistency and transparency in reporting. For further information on Nature Portfolio policies, see our Editorial Policies and the Editorial Policy Checklist.

Statistics

For all statistical analyses, confirm that the following items are present in the figure legend, table legend, main text, or Methods section.

n/a	Confirmed	
☐	☒	The exact sample size (*n*) for each experimental group/condition, given as a discrete number and unit of measurement
☒	☐	A statement on whether measurements were taken from distinct samples or whether the same sample was measured repeatedly
☒	☐	The statistical test(s) used AND whether they are one- or two-sided<br>*Only common tests should be described solely by name; describe more complex techniques in the Methods section.*
☒	☐	A description of all covariates tested
☒	☐	A description of any assumptions or corrections, such as tests of normality and adjustment for multiple comparisons
☐	☒	A full description of the statistical parameters including central tendency (e.g. means) or other basic estimates (e.g. regression coefficient) AND variation (e.g. standard deviation) or associated estimates of uncertainty (e.g. confidence intervals)
☒	☐	For null hypothesis testing, the test statistic (e.g. *F*, *t*, *r*) with confidence intervals, effect sizes, degrees of freedom and *P* value noted<br>*Give P values as exact values whenever suitable.*
☒	☐	For Bayesian analysis, information on the choice of priors and Markov chain Monte Carlo settings
☒	☐	For hierarchical and complex designs, identification of the appropriate level for tests and full reporting of outcomes
☐	☒	Estimates of effect sizes (e.g. Cohen's *d*, Pearson's *r*), indicating how they were calculated

Our web collection on statistics for biologists contains articles on many of the points above.

Software and code

Policy information about availability of computer code

Data collection	We did not use any software for data collection.
Data analysis	We used R version 4.4.1 and Python version 3.10.4 to run GEARS version 0.1.2, scGPT version 0.2.1, scFoundation (build around GEARS version 0.0.2), CPA version 0.8.8, Geneformer version 0.1.0, scBERT from commit hash 262fd4b9 with model weights provided by the authors, and UCE at commit hash 8227a65c. We used igraph version 2.1.4 and locfdr 1.1-8.

For manuscripts utilizing custom algorithms or software that are central to the research but not yet described in published literature, software must be made available to editors and reviewers. We strongly encourage code deposition in a community repository (e.g. GitHub). See the Nature Portfolio guidelines for submitting code & software for further information.

Data

Policy information about availability of data

All manuscripts must include a data availability statement. This statement should provide the following information, where applicable:

- Accession codes, unique identifiers, or web links for publicly available datasets
- A description of any restrictions on data availability
- For clinical datasets or third party data, please ensure that the statement adheres to our policy

Dataset Availability (with link)
Norman Downloaded from scFoundation via Figshare (https://figshare.com/articles/dataset/scFoundation_Large_Scale_Foundation_Model_on_Single-

cell_Transcriptomics_-_processed_datasets/24049200?file=44477939)
Adamson Downloaded via GEARS from Harvard dataverse (https://dataverse.harvard.edu/api/access/datafile/6154417)
Replogle K562 Downloaded via GEARS from Harvard dataverse (https://dataverse.harvard.edu/api/access/datafile/7458695)
Replogle RPE1 Downloaded via GEARS from Harvard dataverse (https://dataverse.harvard.edu/api/access/datafile/7458694)

Research involving human participants, their data, or biological material

Policy information about studies with human participants or human data. See also policy information about sex, gender (identity/presentation), and sexual orientation and race, ethnicity and racism.

Reporting on sex and gender	N/A
Reporting on race, ethnicity, or other socially relevant groupings	N/A
Population characteristics	N/A
Recruitment	N/A
Ethics oversight	N/A

Note that full information on the approval of the study protocol must also be provided in the manuscript.

Field-specific reporting

Please select the one below that is the best fit for your research. If you are not sure, read the appropriate sections before making your selection.

☒ Life sciences ☐ Behavioural & social sciences ☐ Ecological, evolutionary & environmental sciences

For a reference copy of the document with all sections, see nature.com/documents/nr-reporting-summary-flat.pdf

Life sciences study design

All studies must disclose on these points even when the disclosure is negative.

Sample size	We chose publicy available datasets whose experimental designs, incl. sample size, were appropriate as a test case for the proposed method / underlying scientific question.
Data exclusions	We did not exclude any data.
Replication	All material to replicate our results are available at https://github.com/const-ae/linear_perturbation_prediction-Paper/ and https://doi.org/10.5281/zenodo.14833202. However, we did not attempt to independently replicate our results.
Randomization	Not applicable. We present a complete combinatorial matrix of benchmarks of multiple computational methods each applied to multiple datasets and thus could observe each software in all conditions and did not need to randomize software to condition assignment.
Blinding	The analysts were not blinded while evaluating the benchmark. During the implementation of the benchmark, it is not possible to be blinded, as each method requires custom code. And while reporting the results blinding was not necessary as we directly provide the results for metrics that have previously already been used to measure the accuracy of perturbation effect prediction. To ensure that all methods were fairly evaluated, we approached the authors of each method and asked them to scrutinize our implementation.

Reporting for specific materials, systems and methods

We require information from authors about some types of materials, experimental systems and methods used in many studies. Here, indicate whether each material, system or method listed is relevant to your study. If you are not sure if a list item applies to your research, read the appropriate section before selecting a response.

Materials & experimental systems

n/a	Involved in the study
☒	☐ Antibodies
☒	☐ Eukaryotic cell lines
☒	☐ Palaeontology and archaeology
☒	☐ Animals and other organisms
☒	☐ Clinical data
☒	☐ Dual use research of concern
☒	☐ Plants

Methods

n/a	Involved in the study
☒	☐ ChIP-seq
☒	☐ Flow cytometry
☒	☐ MRI-based neuroimaging

Plants

Seed stocks	N/A
Novel plant genotypes	N/A
Authentication	N/A
